# 1936. Efficacy of One Dose of the Respiratory Syncytial Virus (RSV) Prefusion F Protein Vaccine (RSVPreF3 OA) in Adults ≥ 60 Years of Age Persists for 2 RSV Seasons

**DOI:** 10.1093/ofid/ofad500.2467

**Published:** 2023-11-27

**Authors:** Michael G Ison, Alberto Papi, Joanne M Langley, Dong-Gun Lee, Isabel Leroux-Roels, Federico Martinon-Torres, Tino F Schwarz, Richard N Van Zyl-Smit, Susanna Cuadripani, Nancy Dezutter, Olivier Gruselle, Laurence Fissette, Marie-Pierre David, Aurélie Olivier, Marie Van Der Wielen, Dominique Descamps

**Affiliations:** Northwestern University Feinberg School of Medicine, Chicago, IL; University of Ferrara, St. Anna University Hospital, Ferrara, Italy, Ferrara, Emilia-Romagna, Italy; Dalhousie University, IWK Health and Nova Scotia Health, Halifax, Canada, Halifax, Nova Scotia, Canada; The Catholic University of Korea, Seoul, South Korea, Seoul, Seoul-t'ukpyolsi, Republic of Korea; Ghent University and Ghent University Hospital, Ghent, Belgium, Ghent, Oost-Vlaanderen, Belgium; Hospital Clínico Universitario de Santiago, Santiago de Compostela, Spain, Santiago de Compostela, Galicia, Spain; Klinikum Würzburg Mitte, Campus Juliusspital, Würzburg, Germany, Wuerzburg, Bayern, Germany; University of Cape Town and Groote Schuur Hospital, Cape Town, South Africa, Cape Town, Western Cape, South Africa; GSK, Stevenage, Hertfordshire, United Kingdom, Hertfordshire, England, United Kingdom; GSK, Wavre, Belgium, Wavre, Brabant Wallon, Belgium; GSK, Wavre, Belgium, Wavre, Brabant Wallon, Belgium; GSK, Wavre, Belgium, Wavre, Brabant Wallon, Belgium; GSK, Wavre, Belgium, Wavre, Brabant Wallon, Belgium; GlaxoSmithKline Biologicals, Wavre, Brabant Wallon, Belgium; GSK, Wavre, Belgium, Wavre, Brabant Wallon, Belgium; GSK, Wavre, Belgium, Wavre, Brabant Wallon, Belgium

## Abstract

**Background:**

RSVPreF3 OA was recently approved by the United States FDA for the prevention of RSV-related lower respiratory tract disease (RSV-LRTD) in adults ≥ 60 years of age (YOA). We present persistence of vaccine efficacy (VE) of a single RSVPreF3 OA dose, along with VE and safety of annual revaccination dose, over 2 RSV seasons.

**Methods:**

In this phase 3, placebo-controlled, multi-country study (NCT04886596), adults ≥ 60 YOA were randomized 1:1 to receive RSVPreF3 OA or placebo before RSV season 1. RSVPreF3 OA recipients were then re-randomized 1:1 before RSV season 2 to receive a second RSVPreF3 OA dose (RSV_annual group) or placebo (RSV_1dose group); participants who received placebo pre-season 1 received an additional placebo dose (placebo group). VE against first occurrence of RSV-LRTD (confirmatory secondary objectives), severe RSV-LRTD, RSV-LRTD by age, baseline comorbidity and frailty status, and RSV-related acute respiratory illness (ARI) was assessed over 2 seasons. Reactogenicity and safety were also evaluated.

**Results:**

Of 24,973 participants vaccinated before season 1, 24,967 were included in the current VE analyses (RSV_annual: 6,242; RSV_1dose: 6,227; placebo: 12,498). The median follow-up over 2 seasons was 17.8 months. VE of a single dose of RSVPreF3 OA against RSV-LRTD over 2 seasons was 67.2% (97.5% confidence interval [CI]: 48.2–80.0); VE of annual revaccination over 2 seasons was 67.1% (97.5% CI: 48.1–80.0). Sustained VE was observed over 2 seasons against severe RSV-LRTD, against RSV-LRTD among participants 60–69 YOA, 70–79 YOA, those with ≥ 1 baseline comorbidity of interest, pre-frail participants and against RSV-ARI (Figure 1). The reactogenicity (Figure 2) and safety profile of the second RSVPreF3 OA dose was in line with the first dose.

**Figure:**
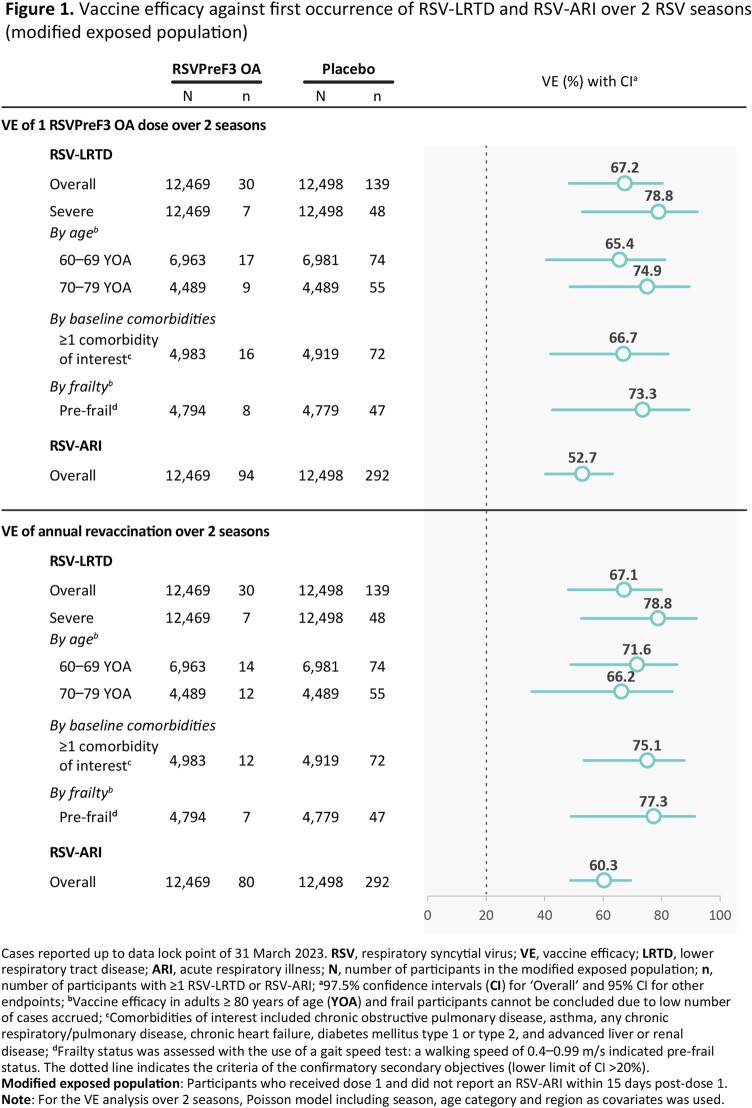

**Figure:**
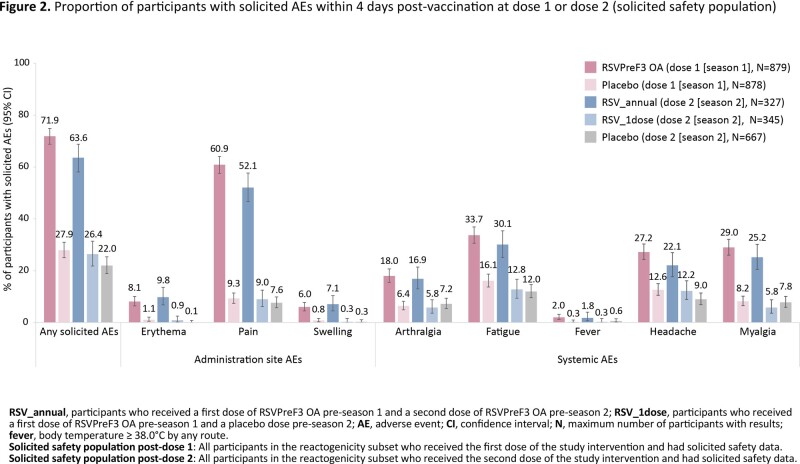

**Conclusion:**

One dose of RSVPreF3 OA is efficacious against RSV-LRTD in adults ≥ 60 YOA over 2 full RSV seasons, as well as against severe RSV-LRTD, and in adults with advanced ages and underlying comorbidities. Revaccination after 1 year does not appear to confer additional efficacy benefit for the overall population. The second RSVPreF3 OA dose had a clinically acceptable safety profile. The clinical development program will further evaluate persistence and the best timing for revaccination.

**Funding:** GlaxoSmithKline Biologicals SA

**Disclosures:**

**Michael G. Ison, MD MS**, Adagio: Advisor/Consultant|Adamis: Advisor/Consultant|Adamis: Board Member|ADMA Biologics: Advisor/Consultant|AlloVir: Advisor/Consultant|AlloVir: Board Member|Atea: Advisor/Consultant|Cidara: Advisor/Consultant|CSL Behring: Board Member|Genentech/Roche: Advisor/Consultant|GSK: Grant/Research Support|Janssen: Advisor/Consultant|Janssen: Board Member|Merck: Board Member|NIH: Board Member|Romark: Advisor/Consultant|Seqiris: Board Member|Shionogi: Advisor/Consultant|Takeda: Advisor/Consultant|Takeda: Board Member|Talaris: Advisor/Consultant|Talaris: Board Member|UpToDate: Stocks/Bonds|Viracor Eurofins: Advisor/Consultant **Alberto Papi, MD**, Agenzia Italiana del farmaco (AIFA): Grant/Research Support|AstraZeneca: Advisor/Consultant|AstraZeneca: Board Member|AstraZeneca: Grant/Research Support|AstraZeneca: Honoraria|Avillon: Advisor/Consultant|Avillon: Honoraria|Chiesi Farmaceutici: Advisor/Consultant|Chiesi Farmaceutici: Board Member|Chiesi Farmaceutici: Grant/Research Support|Chiesi Farmaceutici: Honoraria|EdmondPharma: Honoraria|Elpen Pharmaceuticals: Advisor/Consultant|Elpen Pharmaceuticals: Honoraria|GSK: Advisor/Consultant|GSK: Board Member|GSK: Grant/Research Support|MENARINI: Honoraria|Mundipharma: Honoraria|Novartis: Advisor/Consultant|Novartis: Honoraria|Qivia: Honoraria|Sanofi: Advisor/Consultant|Sanofi: Board Member|Sanofi: Grant/Research Support|Sanofi: Honoraria|Zambon: Honoraria **Joanne M. Langley, MD**, GSK: Grant/Research Support|Inventprise: Grant/Research Support|Merck: Grant/Research Support|Moderna: Grant/Research Support|Pfizer: Grant/Research Support|Sanofi: Grant/Research Support|Seqirus: Board Member|Vaxcyte: Data Safety review board|VBI: Grant/Research Support **Isabel Leroux-Roels, PhD MD**, Curevac: Grant/Research Support|GSK: Board Member|GSK: Grant/Research Support|ICON Genetics: Grant/Research Support|ICON Genetics: Yes|Icosavax: Grant/Research Support|Janssen Vaccines: Advisor/Consultant|Janssen Vaccines: Board Member|Janssen Vaccines: Grant/Research Support|Moderna: Grant/Research Support|MSD: Advisor/Consultant|MSD: Grant/Research Support|OSE Immunotherapeutics: Grant/Research Support|Osivax: Grant/Research Support|Virometrix: Grant/Research Support **Federico Martinon-Torres, MD, PhD, Assoc. Prof**, Abbot: Principal Investigator in randomized controlled trials|Ablynx: Principal Investigator in randomized controlled trials|AstraZeneca: Advisor/Consultant|AstraZeneca: Grant/Research Support|AstraZeneca: Honoraria|Biofabri: Advisor/Consultant|Biofabri: Board Member|Biofabri: Grant/Research Support|Biofabri: Honoraria|GSK: Advisor/Consultant|GSK: Grant/Research Support|GSK: Honoraria|GSK: Clínical trials fees paid to my institution|Janssen: Advisor/Consultant|Janssen: Grant/Research Support|Janssen: Honoraria|Janssen: Clínical trials fees paid to my institution|Medimmune: Principal Investigator in randomized controlled trials|Merck: Grant/Research Support|Moderna: Advisor/Consultant|Moderna: Grant/Research Support|Moderna: Honoraria|MSD: Advisor/Consultant|MSD: attending meetings and/or travel, Principal Investigator in randomized controlled trials|Novartis: Principal Investigator in randomized controlled trials|Novavax: Honoraria|Pfizer: Advisor/Consultant|Pfizer: Board Member|Pfizer: Grant/Research Support|Pfizer: Honoraria|Pfizer: Clínical trials fees paid to my institution|Regeneron: Principal Investigator in randomized controlled trials|Roche: Principal Investigator in randomized controlled trials|Sanofi: Advisor/Consultant|Sanofi: Grant/Research Support|Sanofi: Honoraria|Sanofi: Clínical trials fees paid to my institution|Seqirus: Principal Investigator in randomized controlled trials **Tino F. Schwarz, Prof. Dr. MD**, Alexion: Honoraria|AstraZeneca: Honoraria|Bavarian Nordic: Advisor/Consultant|Bavarian Nordic: Honoraria|Biogen: Honoraria|BioNTech: Board Member|BioNTech: Honoraria|GSK: Advisor/Consultant|GSK: Honoraria|Janssen-Cilag: Advisor/Consultant|Janssen-Cilag: Honoraria|Merck-Serono: Advisor/Consultant|Merck-Serono: Honoraria|Moderna: Advisor/Consultant|Moderna: Honoraria|MSD: Honoraria|Novavax: Advisor/Consultant|Novavax: Honoraria|Pfizer: Honoraria|Roche: Honoraria|Sanofi-Aventis: Honoraria|Seqirus: Advisor/Consultant|Seqirus: Honoraria|Synlab: Honoraria|Takeda: Advisor/Consultant|Takeda: Honoraria|va-Q-tec: Honoraria **Richard N. Van Zyl-Smit, PhD MD**, Boehringer Ingelheim: Grant/Research Support|Boehringer Ingelheim: Honoraria|CIPLA: Honoraria|Glenmark: Honoraria|GSK: Advisor/Consultant|Novartis: Honoraria|OnQsa: Advisor/Consultant **Susanna Cuadripani, MD**, GSK: GSK Employee **Nancy Dezutter, PhD, PharmD**, GSK: Patents still under assessment|GSK: GSK Employee|GSK: Stocks/Bonds|Haleon: Stocks/Bonds **Olivier Gruselle, MS**, GSK: GSK Employee|GSK: Stocks/Bonds **Laurence Fissette, Master in Statistics**, GSK: RSV vaccine patent|GSK: GSK Employee|GSK: Stocks/Bonds **Marie-Pierre David, Master in Statistics**, GSK: GSK Employee|GSK: Stocks/Bonds **Aurélie Olivier, PhD**, GSK: Yes|GSK: GSK Employee|GSK: Stocks/Bonds **Marie Van Der Wielen, MD**, GSK: GSK Employee|GSK: Stocks/Bonds **Dominique Descamps, MD**, GSK: GSK Employee|GSK: Stocks/Bonds

